# Association between otolin-1 and benign paroxysmal positional vertigo: A meta-analysis

**DOI:** 10.3389/fneur.2022.950023

**Published:** 2022-12-15

**Authors:** Xiaoxia Liu, Kun Han, Min Zhou, Yunqin Wu

**Affiliations:** Department of Neurology, Ningbo No. 2 Hospital, Ningbo, China

**Keywords:** BPPV, otolin-1, inner ear proteins, biomarker, meta-analysis

## Abstract

**Background:**

There is increasing research on the potential of inner ear proteins as serum biomarkers for the diagnosis and prognosis of various inner ear diseases. Among them, benign paroxysmal positional vertigo (BPPV) is the most common vestibular disease. Notably, otolin-1, an inner ear-specific protein, is detectable in the serum of most patients with BPPV patients. Therefore, we found a need to conduct this meta-analysis to determine the relationship between otolin-1 in serum and BPPV.

**Methods:**

This meta-analysis was conducted by searching PubMed, EMBASE, Cochrane Library, Google Scholar, and China Network Knowledge Infrastructure databases for the eligible original studies in Chinese or English published between January 2010 and February 2022. Data were collected and pooled by using the mean differences (MDs) corresponding to 95% confidence intervals (CIs). Heterogeneity among these studies was assessed by using I^2^ statistics and the adopted fixed or random-effect mode thereafter. Egger's and Begg's tests were also used to assess the publication bias.

**Results:**

This meta-analysis included six articles with a total of 585 participants. Serum otolin-1 levels were remarkably increased in patients with BPPV as compared to that in healthy controls (MD: 165.38, 95% CI: 110.13–220.64, *p* < 0.00001). However, Egger's and Begg's tests have indicated no publication bias, and the results were reliable based on the sensitivity analysis.

**Conclusion:**

This meta-analysis indicated that there is a higher serum level of otolin-1 in patients with BPPV than in healthy controls. Therefore, otolin-1 may serve as a biomarker for the onset of BPPV.

## Introduction

Benign paroxysmal positional vertigo (BPPV) is the most prevalent peripheral vestibular disease that is characterised by a sudden and transient onset of vertigo (1). It is the most common form of vertigo in older women in their fifties and sixties, with a male-to-female ratio of 1:2.4 (1, 2). Although the clinical symptoms may resolve spontaneously within days to weeks, BPPV has a reported recurrence rate of 7–50% and is associated with anxiety and reduced quality of life (3, 4). Current research asserts that BPPV involves the displacement of the otoconia, whereby the otoliths float into the semicircular canals or attach to the cupula, consequently increasing its sensitivity to gravity (5–7).

The diagnosis of BPPV heavily relies on the clinical history and the elucidation of positional nystagmus on the physical examination (1, 8). However, approximately 30% of BPPV cases are difficult to diagnose and treat, especially in cases of subjective BPPV or multiple canal involvements, which make it difficult to perform diagnostic positional manoeuvres (5, 9). Moreover, in situations where positional nystagmus cannot be elucidated after the patient is sent to the hospital, it is difficult to determine whether the patient is suffering from BPPV. Therefore, the identification of proteins that correspond to BPPV diagnosis may provide significant advances in early diagnosis, timely treatment, and potential prevention of complications.

Otolin-1, a scaffolding protein that is only expressed in the otoconia, vestibule, and cochlear cells, has been reported to be quantifiable in the blood serum (10, 11). It has also been found to increase with age, which could correspond with age-related otoconia degeneration (10, 12, 13). Interestingly, current research shows that otolin-1 levels in serum are elevated in patients with BPPV, indicating its potential as a biomarker for BPPV (10, 14–18).

Therefore, this meta-analysis aimed to review studies in this regard to further clarify the relationship between BPPV and otolin-1.

## Methods

### Study selection

The current review has included English or Chinese studies assessing the relationship between BPPV and otolin-1 levels in serum that were searched from the Cochrane Library, Google Scholar, EMBASE, PubMed, and China Network Knowledge Infrastructure databases between January 2010 and February 2022. The search terms included “otolin-1,” “BPPV,” “benign positional vertigo,” and “benign paroxysmal positional vertigo.” To reduce reviewer bias, two reviewers (KH and XL) carefully evaluated all potentially relevant articles thoroughly during the initial search strategy.

### Inclusion and exclusion criteria

The inclusion criteria were as follows: (1) all patients with BPPV tested for otolin-1, (2) diagnosis of BPPV based on the clinical history of recurrent positional vertigo and typical nystagmus during the Roll or Dix–Hallpike tests, and (3) original case–control studies.

The exclusion criteria were as follows: (1) non-original studies (e.g., case reports), (2) studies without a control group, (3) incomplete data or different study outcomes, and (4) overlapping subjects.

### Data abstraction and quality assessment

Two reviewers (MZ and XL) extracted the following data from each study: the first author's name; publication year; study location and design; method of otolin-1 detection; the number of participants and patients; mean, average, and standard deviation of otolin-1 level; and the research results. The Newcastle–Ottawa Scale was used to assess the quality and the risk of bias of all included studies, ensuring that at the minimum, the articles with intermediate quality were included (19). Furthermore, the study quality was evaluated by the two reviewers (XL and YW), and any disagreements were resolved through discussion.

### Statistical analyses

Mean differences (MDs) or standard mean differences (SMDs) with corresponding 95% confidence intervals (CIs) were used to evaluate the effect size, and study heterogeneity was tested using I^2^ statistics. If *p* ≥ 0.1 or I^2^ ≤ 50% indicated a lack of heterogeneity among studies, a fixed-effect model was used. On the contrary, a random effects model was used if *p* <0.1 or I^2^ > 50%. Sensitivity analyses were also performed based on the rule of omission to analyse heterogeneity. In addition, Egger's and Begg's tests were used to investigate publication bias.

## Results

### Study selection

**Figure 1:**
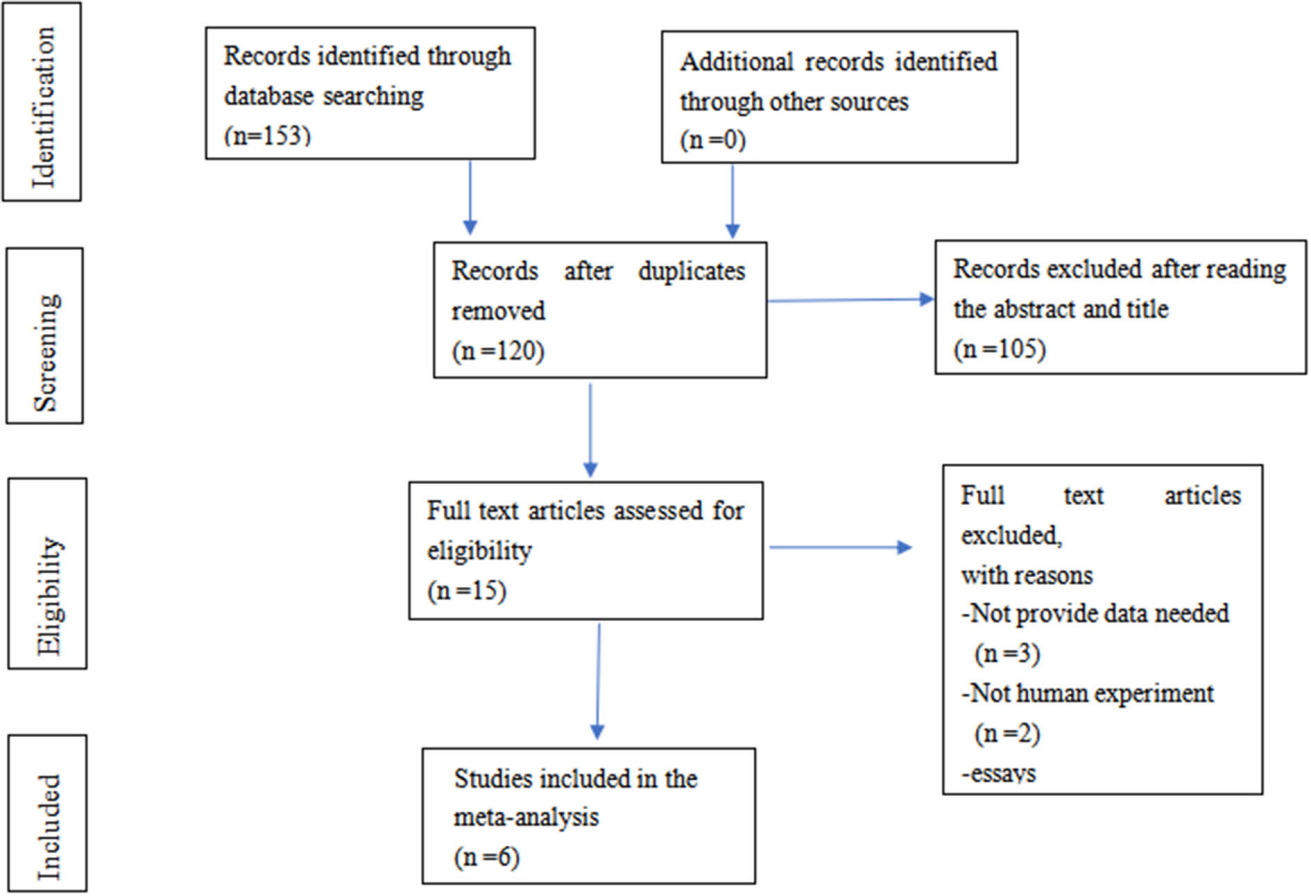
A flow diagram for the selection of studies.

A total of 153 publications were included using the initial search terms. After screening, only six studies with 585 participants were included for further evaluation. The selection process is summarised in Figure 1.

### Study characteristics

The included papers were all the case–control studies that analysed the association between BPPV and otolin-1 (Table 1). These studies were distributed in several regions, including China, India, and the United States (10, 14–18), with 281 patients with BPPV and 304 healthy controls. The clinical characteristics and research conclusions are presented in Table 1.

**Table 1:** Summary of the studies that investigated the association between BPPV and otolin-1 and quality assessment for case–control study.

**First author year**	**Country**	**Group**	**Mean age**	**Case number**	**Otolin-1 (pg/ml)**	**Source of reagents**	**Conclusion**	**NOS**
David, 2021 (14)	India	BPPV Control	46.4 ± 12 47 ± 11.5	40 30	636.8 ± 230.4 236.2 ± 43.5	America	The serum levels of otolin-1 in patients with benign paroxysmal positional vertigo are significantly higher compared with individuals without benign paroxysmal positional vertigo.	5
Harsha, 2021 (16)	India	BPPV Control	46.3 ± 10.6 41.8 ± 10.7	23 23	585 ± 141.5 335.3 ± 61.9	America	Otolin-1 levels are increased and sensitive in BPPV patients, while its specificity needs to be validated.	7
Yun qin, 2020 (15)	China	BPPV Control	62.7 ± 10.7 61.4 ± 11.9	78 121	330.6 ± 76.3 260.1 ± 67.2	China	Serum levels of the otolin-1 protein were significantly higher in patients with BPPV than in healthy controls and may serve as a potential biomarker for BPPV episodes and be used to promote better management of BPPV clinically.	6
Mei yan, 2019 (18)	China	Postmenopausal BPPV Postmenopausal women	63.6 ± 6.7 62.7 ± 6.5	40 40	361.5 ± 186.1 282.6 ± 140	China	Compared to postmenopausal patients without vertigo, the level of otolin-1 increased significantly in postmenopausal BPPV patients.	8
Kourosh, 2014 (10)	USA	Postmenopausal BPPV Postmenopausal women	Not available Not available	14 10	590.3 ± 45 443.1 ± 45.2	America	Inner ear–specific proteins have the potential to serve as biomarkers for otologic disease processes.	6
Shu xia, 2022 (17)	China	Postmenopausal BPPV Postmenopausal women	65.7 ± 10.2 64.6 ± 9.1	86 80	363.6 ± 24.7 270.6 ± 27.1	China	Serum otolin-1 level was higher in postmenopausal BPPV patients compared with postmenopausal controls.	6

### Study findings

**Figure 2:**
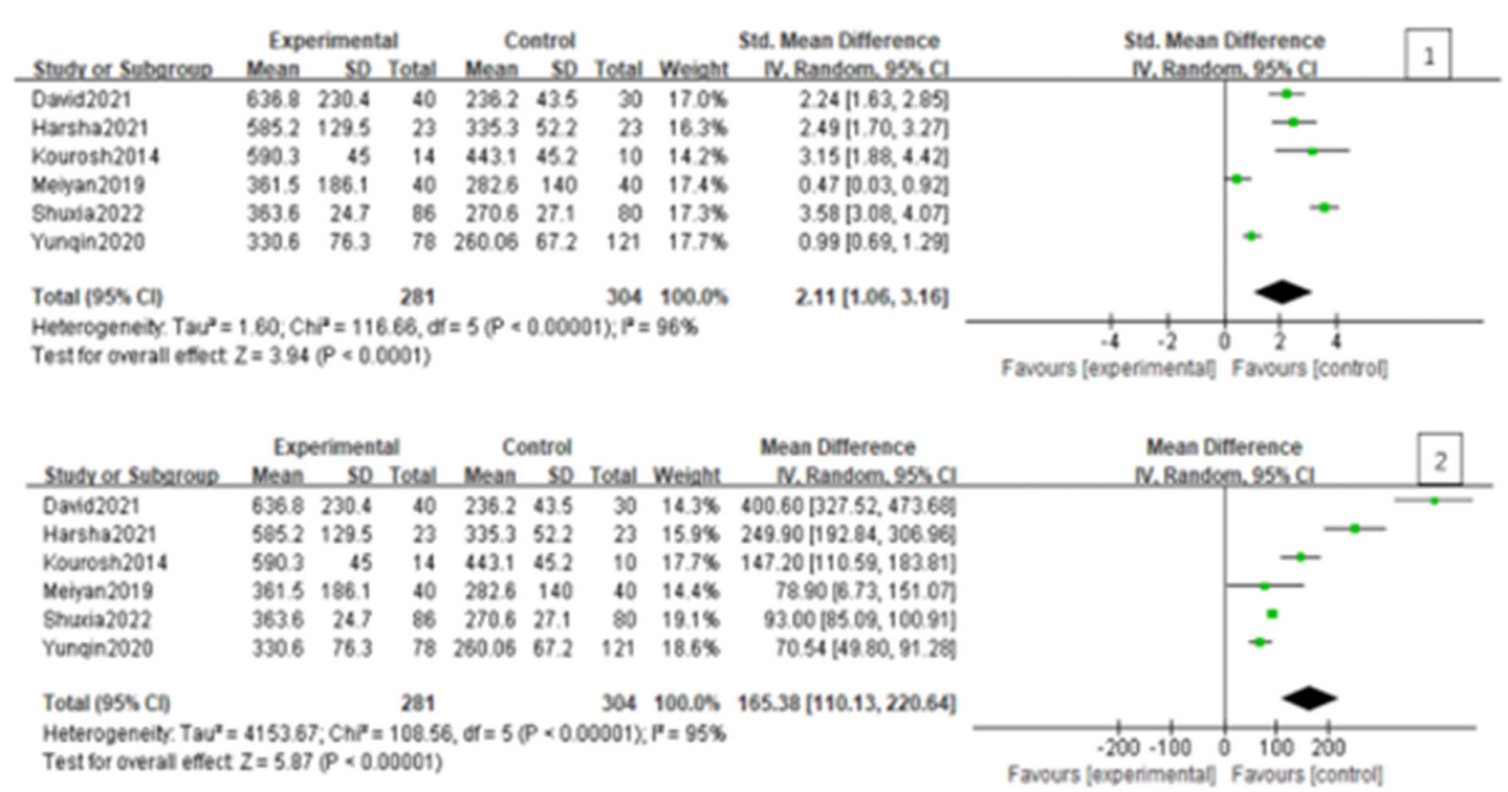
Forest plot of the association between BPPV and otolin-1 using the MD **(1)** and SMD **(2)** effect sizes. And as was showed in the figure, there was a significant association between BPPV and otolin-1 among the two groups.

**Figure 3:**
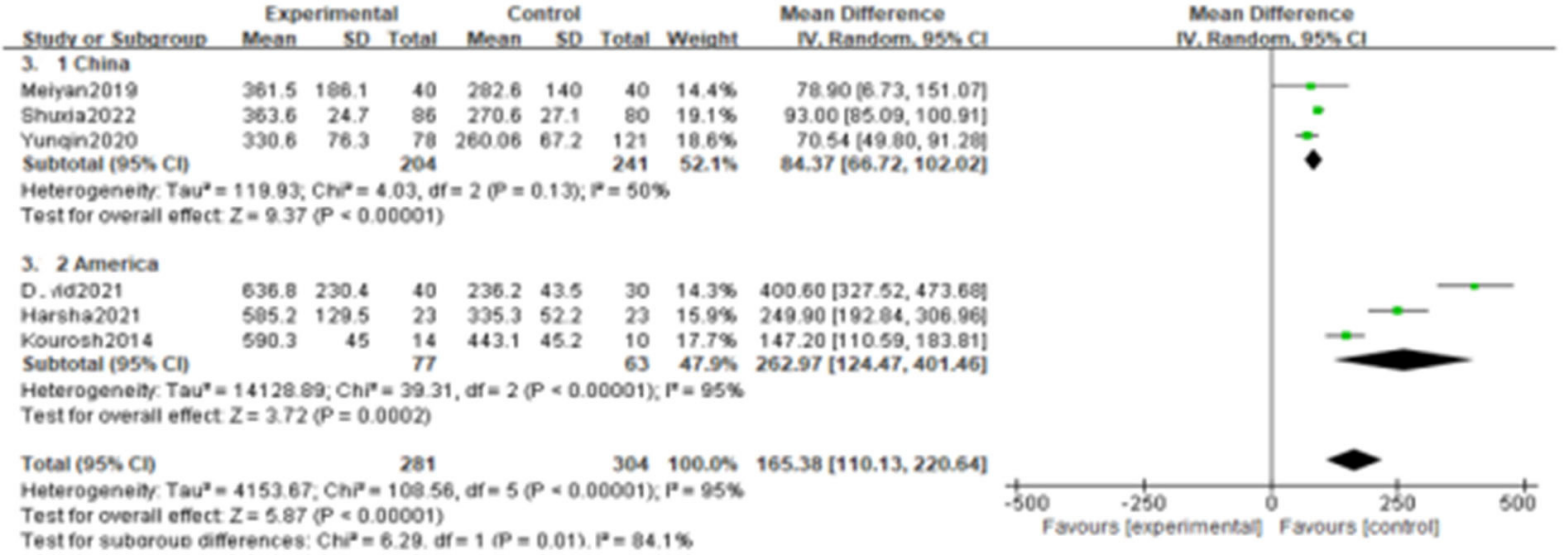
Subgroups analysis was performed according to the reagent sources from China and American.

**Figure 4:**
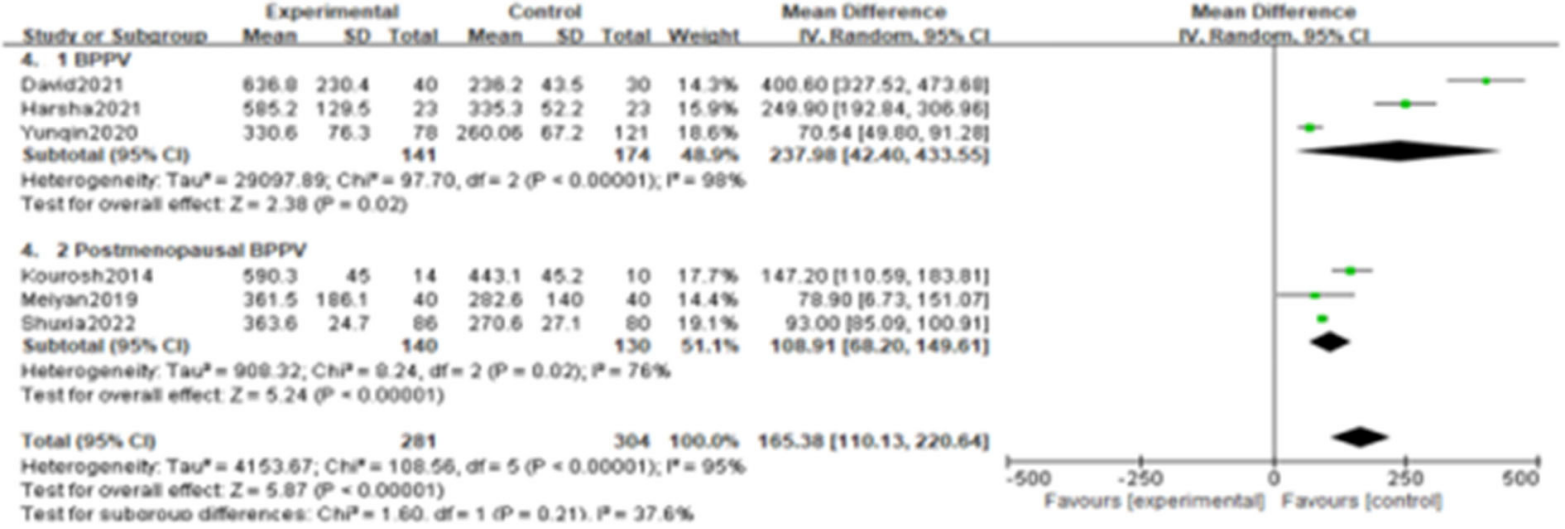
Subgroups analysis was performed according to differences in study populations.

Since the heterogeneity among the included studies was high (I^2^ = 95%), the random-effects model was used (Figure 2). All six studies showed a significant association between BPPV and otolin-1 in terms of both MD (Figure 2.1, MD: 165.38, 95% CI: 110.13–220.64, *p* < 0.00001) and SMD effect size (Figure 2.2, SMD: 2.11, 95% CI: 1.06–3.16, *p* < 0.00001). Subgroup analysis based on the source of reagents and menopause was then performed to identify sources that may have contributed to heterogeneity. In this case, heterogeneity decreased significantly after subgroup analysis (Figures 3, 4; I^2^ = 50%, I^2^ = 76%), indicating that reagents and oestrogen levels could be the primary source of heterogeneity.

### Publication bias and sensitivity analysis

**Figure 5:**
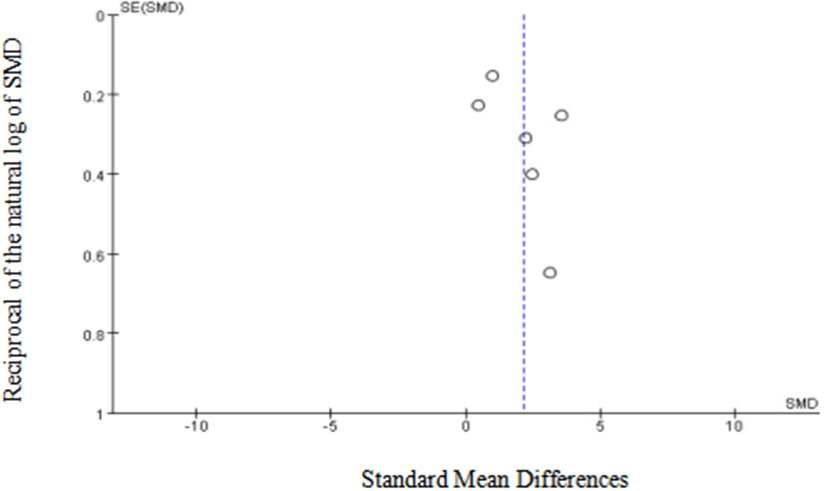
Funnel plot of the included studies in the meta-analysis. X-axis represents SMD, y-axis represents reciprocal of the natural log of SMD and the circles represent the included studies.

**Figure 6:**
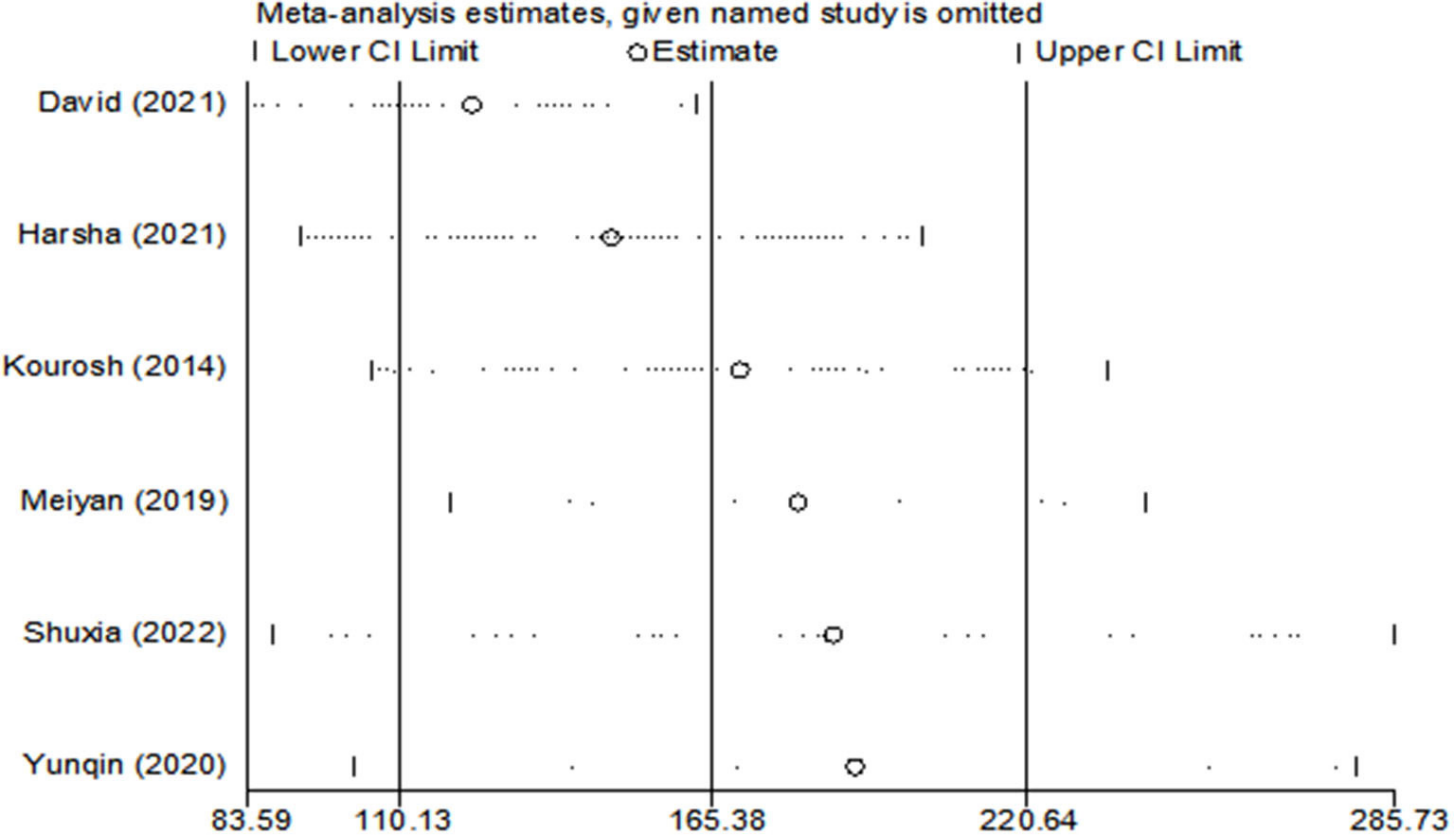
Sensitivity analysis of the included studies using leave-one-out analyses.

Egger's (*p* = 0.15) and Begg's tests (*p* = 0.13) indicated little to no publication bias. Similarly, funnel plot analysis showed minimal publication bias (Figure 5). Sensitivity analysis was also conducted using leave-one-out analyses and changing effect size. In addition, the results of the previous analyses were not altered by the exclusion of any articles, indicating good reliability and stability (Figure 6).

## Discussion

In this meta-analysis, we assessed the relationship between BPPV and otolin-1 levels in serum, which showed higher levels of otolin-1 in patients with BPPV as compared with healthy controls. As such, otolin-1 may be a potential biomarker for diagnosing BPPV episodes.

Benign paroxysmal positional vertigo is the most common cause of vertigo, which is characterised by recurrent episodes that are triggered by changes in head position (5, 20). Although BPPV is benign and has a high rate of resolution, it affects the quality of life as patients report marked limitations in their daily activities (1, 21, 22). This is due to a high relapse rate of 7–50% (3, 4), which consequently causes anxiety, reduces the quality of life, and increases the risk of fall from instability (23, 24). Furthermore, BPPV comprises 24.1% of all clinical visits due to dizziness (2), and its medical burden is also known to be high (20, 25).

Most BPPV cases do not have a clear aetiology, with 75% of them being diagnosed as idiopathic (12, 26). Although the underlying pathophysiology of BPPV is yet to be fully elucidated, the main causes of BPPV have been proposed to be due to the degeneration of otoliths and displacement in the semicircular canal (6, 27). Otoconia consist of calcium carbonate deposits on a protein framework composed of otolin-1 and otoconin 90 (6, 11). The presence of calcium carbonate particles in the semicircular canal makes the canal sensitive to gravity, resulting in the dysfunctional sensation of angular acceleration (28). Thus, even if the current treatment strategies aim to minimise the impact of displaced otoconia for symptomatic relief, they do not address the degeneration of utricular otoconia, resulting in frequent recurrences.

From the clinical point of view, circulating biomarkers for otologic diseases are significant indicators for the investigation of inner ear damage, the diagnoses of inner ear disorders, tracking of treatment response, and the development of new treatments (11). Laboratory biomarkers should therefore be explored as tools to achieve a faster and more accurate diagnosis of BPPV, wherein otoconial degeneration may be utilised as a diagnostic modality (14, 25). Several recent studies have shown that inner ear-specific proteins are present in the blood serum and can be quantified (10, 29, 30). According to the literature search, eight proteins exclusive to the inner ear were identified, among which prestin and otolin-1 can be detected in the circulation (30). Prestin, a motor protein found in the lateral membrane of the outer hair cells (31), is known as a potential biomarker of noise- and drug-induced ototoxicity (10, 29, 32, 33).

Otolin-1 is a 70-kilodalton glycoprotein that acts as a scaffolding protein by attracting otoconial core matrix proteins and otoliths to the acellular gel matrix and inner ear sensory epithelia (11). Although otolin-1 exits in the endolymph, it can cross the labyrinth-blood barrier (10, 34), thus its detection and measurement in the peripheral blood are possible. Moreover, otolin-1 has been found to increase with age in healthy individuals and is significantly elevated in patients over 65 years or patients after mastoidectomy (12). This is consistent with previous scanning electron microscopy findings of age-related otoconial degeneration and reports of increased BPPV prevalence with age (6, 35, 36), making it a useful biomarker for the degeneration of otoconia (12). Elevated otolin-1 levels have also been reported in patients with BPPV, providing evidence for its potential as a biomarker for inner ear conditions and for predicting the risk of BPPV in the general population (17). Furthermore, studies have reported on the association between elevated serum otolin-1 levels and increased risk of BPPV relapse (25). The mechanism that the otolin-1 serum level is elevated in patients with BPPV is unclear. Some studies have shown that the level of otolin-1 is associated with calcium dysregulation (16, 37). From a practical standpoint, otolin-1 may be diagnostic in the management of the remaining 30% of challenging BPPV cases (10). The mechanism explaining why the otolin-1 serum level is elevated in patients with BPPV remains unclear. Further studies should be conducted with larger patient cohorts and dynamic assessments of otolin-1 levels in different stages to establish its value. Furthermore, suitable BPPV models should be established to study these metabolic processes in the near future.

Despite these findings, this meta-analysis has several limitations. First, the included papers were case–control studies, which are less credible than randomised controlled trials. Second, there was significant statistical heterogeneity among the studies. Owing to the small sample size and the fact that some of the studies were conducted on postmenopausal women, subgroup analysis by assay and menstruation status partially eliminated heterogeneity. This indicates that the reagents, oestrogen level, and age may have caused the heterogeneity in the meta-analysis. Third, language bias may have been present, since the studies were limited to those written in Chinese or English. Finally, this study is still in the initial phase. Since there was no identical standard for otolin-1 testing and no mention of the time from the symptom onset to blood sample collection, these nuances may have led to the differences in otolin-1 levels.

## Conclusion

Serum otolin-1 levels in patients with BPPV were significantly higher than that of healthy controls and may serve as a biomarker for the diagnosis of BPPV episodes. Since the study is still in its initial phase, further investigations with larger sample sizes over extended periods should be conducted to monitor changes in blood otolin-1 levels, as well as facilitate the early identification and prevention of BPPV.

## Data availability statement

The original contributions presented in the study are included in the article/supplementary material, further inquiries can be directed to the corresponding author.

## Author contributions

XL and MZ have acquired and analysed the data and drafted the manuscript. MZ and KH have acquired and analysed the data and revised the manuscript. YW has conceptualised and supervised the study and revised the manuscript. All authors contributed to the article and approved the submitted version.
